# Under-Reporting of Human Leptospirosis Cases in Cities of Triângulo Mineiro, Minas Gerais, Brazil

**DOI:** 10.3390/tropicalmed9100229

**Published:** 2024-10-04

**Authors:** Mariani Borges Franco, Lara Reis Gomes, Cristina Rostkwoska, Ana Cláudia Arantes Marquez Pajuaba, José Roberto Mineo, Anna Monteiro Correia Lima, Stefan Vilges de Oliveira

**Affiliations:** 1 Graduate Program in Health Sciences, Federal University of Uberlândia, Uberlândia 38400-902, MG, Brazil; marianiborges@ufu.br; 2 Laboratory of Contagious Infectious Diseases, Faculty of Veterinary Medicine, Federal University of Uberlândia, Uberlândia 38400-902, MG, Brazil; larareis@ufu.br (L.R.G.); annalima@ufu.br (A.M.C.L.); 3 Laboratory of Immunoparasitology, Institute of Biomedical Sciences, Federal University of Uberlândia, Uberlândia 38400-902, MG, Brazil; cristina.rostkwoska@ufu.br (C.R.); ana.pajuaba@ufu.br (A.C.A.M.P.); jrmineo@ufu.br (J.R.M.); 4 Department of Collective Health, Faculty of Medicine, Federal University of Uberlândia, Uberlândia 38400-902, MG, Brazil

**Keywords:** acute febrile illness, zoonosis, dengue, *Leptospira*, leptospirosis

## Abstract

Leptospirosis is an infectious disease caused by the pathogenic *Leptospira* species through direct or indirect contact with infected animals. Due to protean clinical manifestation in the early stages, leptospirosis is often difficult to distinguish from other common acute febrile illnesses, such as dengue. Thus, this study aimed to investigate the prevalence of leptospirosis in suspected dengue patients whose serological diagnosis was negative. A total of 449 serum samples from patients (negative IgM-ELISA dengue) with fever, headache, myalgia, and nausea were tested. The Dual-Path Platform (DPP) rapid test developed by the Instituto de Tecnologia em Imunobiológicos Bio-Manguinhos in the city of Rio de Janeiro, Brazil was used for screening IgM antibodies against *Leptospira* in blood serum, and the microscopic agglutination test (MAT) was performed on samples positive in the DPP for leptospirosis, as well as on an equal number of negative samples. Results: The data obtained from the samples analyzed with the DPP assay showed 26 positive results (5.79%), of which 38.46% were male and 61.54% female, with a mean age of 41 years. We tested 52 samples using the MAT, including 26 reactive for IgM and 26 non-reactive in the DPP assay. Nine samples (17.31%) were reactive, and among them, six also showed reactivity in the DPP assay. Of the six samples reactive in both tests, 66.67% were female, living in urban areas in the city of Uberlândia, with a mean age of 50 years, being 50% white, 33.33% brown, and 16.67% black. The findings demonstrated that leptospirosis cases are underdiagnosed and undertreated in the study population and more attention needs to be paid for ruling out leptospirosis and other pathogens causing acute febrile illness in dengue-endemic areas.

## 1. Introduction

Leptospires are a group of spiral-shaped bacteria within the order Spirochaetales, which cause leptospirosis, an acute febrile illness of sudden onset. Leptospires exhibit high genetic diversity and can be classified based on antigenic differences on their cell surface (serovars). Of the genus *Leptospira*, 10 (ten) pathogenic species are known with more than 300 serovars [1]. Different serovars are associated with different hosts and environments. Some groups, such as the Icterohaemorrhagiae serogroup, are associated with rodents and can cause leptospirosis in humans [1].

Leptospirosis has a clinical spectrum ranging from mild or asymptomatic symptoms to more severe forms. The infection is an important global cause of acute fever and a leading cause of morbidity among zoonotic diseases, transmitted by domestic and wild synanthropic animals, with rodents being the principal disseminators. Other reservoir hosts include dogs, pigs, cattle, horses, sheep, and goats. These animals can harbor leptospires in their kidneys and excrete them via urine into the environment, contaminating water, soil, and food [2].

Leptospirosis is primarily transmitted to humans through direct contact with an infected animal or indirect contact with the environment, such as soil and water contaminated with body fluids (especially urine) of infected animals, where leptospires can penetrate intact skin and mucous membranes. Less commonly, contamination can occur through contact with infected animals’ blood, tissues, and organs, or by ingesting leptospiral-contaminated water or food [3].

After an incubation period of 5 to 14 days, symptoms such as fever, headache, myalgia, fatigue, nausea, and vomiting may appear, known as the leptospiremic phase. The disease can progress to the late stage with severe and, sometimes, fatal manifestations in approximately 15% of patients. The severe form, recognized as Weil’s syndrome, is characterized by jaundice, renal failure, and hemorrhage. This highlights the importance of early diagnosis to initiate treatment since the progression to severe form has a high fatality rate. In Brazil, an annual average of 3600 cases and 375 deaths has been confirmed [4]. Due to the similarity of clinical signs in the early stage of leptospiral infection, leptospirosis is difficult to distinguish from other acute febrile illnesses, including yellow fever, viral hepatitis, and dengue [3,5,6].

Dengue is the most prevalent arthropod-borne viral disease in Brazil. The dengue virus (DENV) is transmitted through the bite of the Aedes aegypti mosquito. The period of highest transmission occurs during the rainy months in each region. According to the Brazilian Ministry of Health, 1,544,987 cases were notified in 2019, with 782 deaths. Minas Gerais, São Paulo, and Goiás were the states with the highest number of the cases [7].

The differential diagnosis of leptospirosis and dengue remains a major challenge for medical care and surveillance programs. As both diseases have similar clinical profiles and seasonal onset, dengue (the disease with higher incidence) is often considered the most probable diagnosis in patients with acute febrile illness in endemic areas, which may underestimate leptospirosis burden [8].

Thus, this study aimed to investigate the prevalence of antibodies against pathogenic *Leptospira* species in patients with acute febrile illness referred for dengue diagnosis who tested negative serologically.

## 2. Materials and Methods

**Figure 1:**
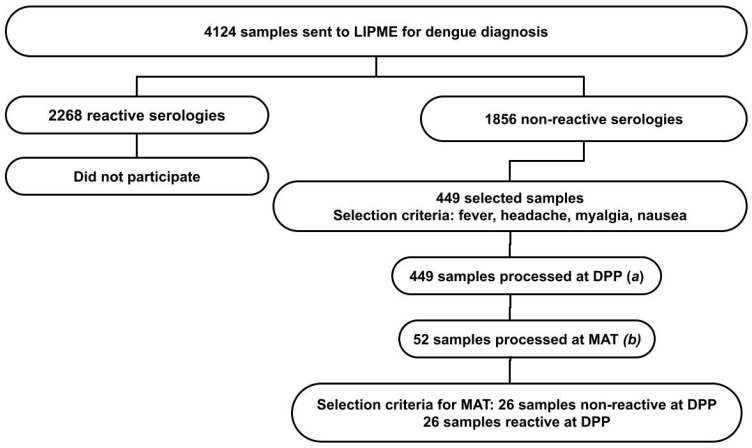
Prevalence of leptospirosis in suspected dengue patients with negative serological diagnosis. Criteria for sample selection and serological diagnostic methods for leptospirosis: (**a**) Dual-Path Platform (DPP) immunochromatographic rapid test for leptospirosis *n* = 449; (**b**) microagglutination test (MAT) for leptospirosis, *n* = 52.

We analyzed epidemiological data from 4124 patients referred to the Laboratory of Immunoparasitology Dr. Mário Endsfeldz Camargo (LIPME) at the Federal University of Uberlândia for dengue diagnostic testing in the year 2019. Through convenience sampling, 1856 aliquots of blood serum were selected, with negative results for dengue based on the results of the Dengue IgM Capture ELISA (PanBioTM, Abbott, St. Ingbert, Germany). Among these, 449 samples were subjected to serological analysis for leptospirosis due to specific symptomatology (Figure 1).

No patient recruitment was conducted, relying solely on previously recorded secondary data in the institution’s database. All collected information was identified using an internal control number. We organized the epidemiological data available at the LIPME for analysis and sample selection. The following data were collected through an epidemiological record completed by healthcare professionals: municipality, area of residence, age, sex, race, skin color, occupation, education level, onset date of symptoms, and collection date. Clinical data comprised signs and symptoms of fever, myalgia, headache, rash, vomiting, nausea, back pain, conjunctivitis, arthritis, arthralgia, petechiae, leukopenia, retro-orbital pain, pre-existing conditions, and information on case progression: recovery, hospitalization, hospital admission date, and date of death. The notification form was assessed for completeness for suspected dengue cases by healthcare professionals. An exploratory descriptive analysis of the data was carried out. The data are presented as absolute frequencies, relative frequencies, and measures of central tendency. The results are presented in tables to facilitate visualization.

LIPME collaborates with the Regional Health Coordination of Uberlândia for dengue diagnosis in the Triângulo Mineiro, which is one of the ten planning regions in the Minas Gerais state, southeastern Brazil. The samples included in this study were derived from suspected dengue cases of the following municipalities: Uberlândia, Araguari, Monte Carmelo, Monte Alegre de Minas, Patrocínio, Grupiara, Coromandel, Nova Ponte, Abadia dos Dourados, Cascalho Rico, Iraí de Minas, Romaria, Douradoquara, Araporã, Estrela do Sul, and Prata.

### 2.1. Dual-Path Platform (DPP) Immunochromatographic Rapid Test for Leptospirosis

We selected 449 serum samples from patients who had a negative IgM-ELISA for dengue in 2019 and with the following clinical symptoms in the epidemiological records: fever, headache, myalgia, and nausea. The sampling prioritized these symptoms reported by the patient to the healthcare professional, as they are also characteristic symptoms of leptospirosis according to the Ministry of Health and *the Merck Manual* [9,10]. Samples lacking epidemiological data or whose serum was not in good storage condition for serological processing (samples with hemolysis, lipemia, insufficient quantity, and in poor condition) were not selected.

We performed the rapid immunochromatographic test for qualitative screening of specific IgM antibodies for *Leptospira* in human serum, plasma, or whole blood, developed by the Oswaldo Cruz Foundation (Fiocruz) at the Immunobiological Technology Institute (Bio-Manguinhos, Rio de Janeiro, Brazil).

The DPP rapid test (DPP Leptospirosis—Fiocruz) was designed based on recombinant proteins. These native proteins are present on the *L. interrogans* serovar Copenhageni surface and the assay uses a combination of two colloidal gold particles conjugated with a human protein and anti-IgM. For sample processing, we followed the kit manufacturer’s recommendations. The detection of two pink/purple lines, one in the control area and the other in the test area, indicates a positive result. The intensity of the test lines may vary depending on the concentration of specific antibodies. The manufacturer’s guidelines state that even a very clear line should be considered a reactive result. All samples that showed positive results were repeated to confirm the outcomes.

The presence of only one purple/pink line indicates a non-reactive test, thus suggesting the absence of anti-*Leptospira* antibodies. However, it does not exclude the possibility that the individual who has had recent contact with the specific antigen has the *Leptospira* infection due to the immunological window.

### 2.2. Microagglutination Test (MAT)

The MAT is a highly accurate test for epidemiologic studies, strain identification, assessing probable infecting serogroups, and confirming illness for public health surveillance. The MAT remains the reference test and is used to detect antibodies and determine their titer. We performed the microagglutination test (MAT) for positive samples in the leptospirosis screening test. The test determines agglutinating antibodies (IgM and IgG) against the bacteria *Leptospira* spp. in the patient’s blood serum by mixing different serovars of the bacterium in serial dilutions with the test serum. Antileptospira antibodies present in the serum cause the agglutination of leptospires. It is the serological technique recommended by the World Health Organization (WHO) as the standard test for diagnosing leptospirosis [11].

We performed the microagglutination test (MAT) for 26 DPP positive samples and an equal number of randomly selected 26 DPP negative samples as it is not routinely used for diagnosis in febrile patients.

To perform the MAT, the following serogroups and serovars were tested: Australis (*L. interrogans* serovar Bratislava), Ballum (*L. borgpetersenii* serovar Castellonis), Canicola (*L. interrogans* serovar Canicola), Djasiman (*L. interrogans* serovar Djasiman), Grippotyphosa (*L. kirschneri* serovar Grippotyphosa), Hebdomadis (*L. interrogans* serovar Hebdomadis), Icterohaemorragiae (*L. interrogans* serovar Copenhageni; *L. interrogans* serovar Icterohaemorragiae), Pomona (*L. interrogans* serovar Pomona), Sejroe (*L. interrogans* serovar Hardjoprajitino; *L. interrogans* serovar Wolffi), and Tarassovi (*L. borgpetersenii* serovar Tarassovi). Every serum that agglutinates at least 50% of the leptospires at a 1:100 dilution is considered seroreactive. Positive and negative controls were included in all tests. Seroreactive samples at screening were titrated (1:200; 1:400; 1:800; 1:1600; 1:3200). The serovars were maintained by the Laboratory of Infectious Contagious Diseases (LADOC) at the Faculty of Veterinary Medicine of the Federal University of Uberlândia.

This study was approved by the Research Ethics Committee of the Federal University of Uberlândia (UFU) under protocol number 5,392,382 and followed the guidelines of the National Research Ethics Commission (CONEP) for research involving humans (Resolutions No. 466 of 12 December 2012, and No. 510 of 7 April 2016 of the National Health Council).

## 3. Results

A total of 449 patients were included according to the selection criteria of this study, 148 male and 301 female, with a mean age of 43 years, and 39.42% were aged between 41 and 60 years. Among the participants, 98.22% were from the urban area. They were referred to the dengue diagnosis service predominantly in February and March 2019. The main clinical complaints besides those used for the inclusion criteria (fever, myalgia, headache, and nausea) were backache (54.12%), retro-orbital pain (52.34%), and vomiting (51.22%).

Of the samples analyzed using the DPP assay, 26 were positive (5.79%). The average collection time for reactive samples was 7 days, with a maximum of 12 days and a minimum of 5 days. Of the 26 reactive samples, 38.46% were male (10 samples) and 61.54% (16 samples) were female, with one of the patients in the first trimester of pregnancy. Positive individuals had a mean age of 41 years and were residents of Uberlândia (69.23%), Araguari (7.69%), and Monte Carmelo (23.08%), predominantly in urban areas (92.31%), who reported brown race/skin color (46.15%). After the signs and symptoms used as selection criteria, backache and retro-orbital pain were the clinical complaints most reported by patients (57.69%), followed by vomiting (38.46%) and severe arthralgia (34.62%). Only 3.85% of the patients had pre-existing diseases (diabetes and arterial hypertension). Hospitalization occurred in 23% of the reactive cases in the DPP assay (six patients) (Table 1). The hospitalized patients had postural hypotension, mucosal bleeding/other hemorrhages, abrupt drop in platelets, hepatomegaly, and abdominal pain. One of the patients showed a small unilateral effusion on thoracic tomography.

Of a total of 52 samples (26 positive and 26 negative in DPP test) subjected to the MAT, 17.31% (9 samples) were positive, and 11.5% (6 samples) were positive in both the DPP test and the MAT. Of the six positive samples in both tests, 66.67% were female, living in urban areas of Araguari (33.33%) and Uberlândia (66.67%), with a mean age of 50 years, who classified themselves as white (50%), brown (33.33%), and black (16.67%).

Serum samples from nine patients showed reactions for at least 10 serovars (Canicola, Tarassovi, Grippotyphosa, Djasiman, Hardjoprajitino, Hebdomadis, Icterohaemorragiae, Pomona, Bratislava, and Copenhageni), according to the agglutinating titers depicted in Table 2.

The positive patients according to both serological techniques were not hospitalized and didn’t show severe symptoms. One of them had hypertension and diabetes and, in addition to fever, myalgia, nausea, and headache, had rash, arthralgia, petechiae, and retro-orbital pain. Notably, one patient who was reactive in the MAT and non-reactive in the DPP assay was hospitalized and had pre-existing lesions.

## 4. Discussion

A significant frequency of reactive serological samples for leptospirosis (5.79%) was found in patients with acute febrile illness referred for dengue diagnosis through the DPP assay, inferring a possible underestimation of leptospirosis. All patients who tested positive for leptospirosis were residents of urban areas. A previous study carried out in the São Paulo state demonstrated a prevalence of 1.04% of leptospirosis cases using the MAT in patients also referred for dengue diagnosis [12].

A study conducted in the city of Uberlândia reported that human leptospirosis cases may be underestimated, as the authors identified 28.4% of domestic dogs reactive for leptospirosis, mainly to Autumnalis (34.2%) and Tarassovi (23.7%) serovars. As the samples were collected from asymptomatic dogs (probably harboring the bacteria asymptomatically) and randomly during an anti-rabies vaccination campaign, the authors believe that the dogs would serve as reservoirs of the disease, being able to infect humans and maintain the epidemiological chain of the agent (the same serovar was found in human leptospirosis cases). In fact, dogs play a major role in the transmission of the disease to humans, harboring leptospires for a long period in the kidneys and being able to eliminate them in the urine without showing clinical signs or after obtaining clinical improvement. Furthermore, the official number of human leptospirosis cases was lower than that found in the records of laboratories of the city [13]. In the study region, the rainy season begins in October and ends in April. Most dengue and leptospirosis cases are concentrated during this period. As our sampling was concentrated at the end of the rainy season, our data may still be underestimated.

It is important to remember that high titers are detectable in the MAT from the second week of illness [14]. These serovars should not be excluded from a list of suspected infecting serovars. In this study, higher MAT titers were observed against the serovar Canicola in two patients (800 titer), and other serovars (400), which is extremely concerning. This is especially true because a *Leptospira* agglutination titer of ≥200 in the MAT may indicate *Leptospira* spp. infection, particularly when combined with clinical signs and epidemiological history. Additionally, previous studies have shown a positive correlation between asymptomatic seropositive dogs and human leptospirosis [15].

In two Brazilian biomes, Pantanal and Caatinga, the prevailing serovars for MAT responses were as follows: Bratislava (in dogs, wild animals, and humans); Grippotyphosa (in cattle and horses); Copenhageni (in dogs and humans); Patoc (in cattle and horses); and Panama (in goats and sheep). A previous study confirmed that not only dogs, but also free-living wild species, serve as reservoirs of leptospires for domestic animals and humans [16].

In the state of Rio de Janeiro, out of 51 volunteers, 25.5% tested positive for various *Leptospira* serovars. The predominant serovars found were Hebdomadis (92%), Patoc (69.23%), and Pomona (53.85%). The high prevalence of less pathogenic serovars indicates a high risk of severe leptospirosis if pathogenic serovars are introduced by migrating infected rodents [17].

Although the MAT is the gold standard for serodiagnosis of leptospirosis, all tests have their limitations. The MAT’s performance depends on the strains used and the quality of the antigen suspensions, where 36% of confirmations may need a paired sera for comparison of titers; its sensitivity depends on the days of disease progression, being lower in the acute stage (first week) [18,19]. The MAT result indicates the presence of antibodies against multiple serovars of *Leptospira*. Patients were infected with more than one serovar, highlighting the importance of avoiding direct or indirect contact with infected animals, as humans are accidental hosts. Few studies have investigated hospital and intensive care unit admissions and the clinical progression of patients [20]. Most cases of leptospirosis are mild, but 5 to 15% can be severe and fatal, mainly due to misdiagnosis, inappropriate treatment, or the pathogenicity of certain serogroups. In our study, we could not find out whether the patients we identified as positive for leptospirosis had the same diagnosis when dengue was ruled out. Therefore, we do not know if the clinical and therapeutic approaches were appropriate for leptospirosis. Therefore, the number of reactive samples in our study could be greater since the tested samples had been collected, on average, 7 days after the onset of symptoms. There was no possibility of obtaining a second sample to perform a paired test and comparison of titers, because the titers initially identified in the MAT may be from previous contact with *Leptospira*. This fact justifies the finding of three samples showing agglutination in the non-reactive DPP assay.

The DPP test used showed a sensitivity of 81.5% [21] in samples collected between 7 and 14 days, demonstrating the usefulness of the screening test. A recent study that examined its diagnostic accuracy and clinical utility using finger stick blood, venous whole blood, and serum revealed that the DPP assay is accurate, portable, and reliable for early diagnosis of human leptospirosis, which is essential to initiate treatment, as the progression of leptospirosis in more severe cases is rapid and has a high case fatality rate.

In Brazil, according to the Notifiable Diseases Information System (SINAN), 3709 cases and 325 deaths from leptospirosis were confirmed in 2019, with 189 cases and 19 deaths in Minas Gerais. According to the Brazilian Ministry of Health data released by the Information Technology Department of the Unified Health System (DATASUS), from 2010 to 2019, 42 leptospirosis cases were confirmed in the municipalities served by the LIPME. There are records of the disease in all federation units, with a greater number of patients in the south and southeast regions. Importantly, leptospirosis has an average case fatality rate of 9%. In turn, 2019 was considered an epidemic year for dengue, according to the Epidemiological Bulletin of the Health Surveillance Secretariat of the Ministry of Health, with more than 20,000 severe cases and a case fatality rate of 0.05% [22].

Comparing both diseases, dengue receives more government attention, and diagnostic confirmation is sought more frequently than for leptospirosis. Despite the international recognition of leptospirosis as a Neglected Tropical Disease (NTD), Martins and Spink (2020) explain some reasons why the infection still does not receive due attention from public policies in Brazil. Investments in research and interventions are defined using epidemiological, demographic, and clinical data and social impacts. Moreover, epidemiological and demographic data may be non-existent, non-specific, or inaccurate. Thus, this information becomes invisible [23]. In our study, the incompleteness of the epidemiological data in the analyzed notification forms was verified, mainly regarding occupation (55.46%), race/skin color (8.91%), education (40.98%), and autochthony (70.16%), compared to the total samples. The low completeness of reporting forms can harm the understanding of the epidemiological profile of the disease. Therefore, the training of health professionals should be performed to address this issue and value the importance of correctly recording cases.

The clinical data for leptospirosis can mimic the signs and symptoms of other diseases, making it difficult to recognize or diagnose, especially during the acute phase. Moreover, the lack of specific symptoms and appropriate diagnostic methods and the passive nature of surveillance programs may lead to an underestimation of the burden of leptospirosis [8]. Therefore, all acute febrile illnesses in these endemic areas can be subjected to a panel of tests, particularly dengue and leptospirosis. Future studies should also analyze positive dengue samples, thus seeking to identify possible co-infections (dengue and leptospirosis) that may be occurring in this territory without proper diagnosis.

Human leptospirosis has still been related to individuals who live in rural areas [24,25]. However, in this study, patients with suspected dengue who were positive in serological tests for leptospirosis mostly lived in urban areas. This result emphasizes the reasons for the lack of initial suspicion and, consequently, negligence in the correct diagnosis of leptospirosis.

Remarkably, our findings showed that the diagnosis of leptospirosis is being under-reported. This is alarming, as the treatment may have been insufficient since, for leptospirosis patients, specific antimicrobials are required, which are not used in cases of viral infection. We believe that a greater number of leptospirosis cases could have been found if there had been adequate clinical suspicion and the possibility of performing a greater number of tests in patients with acute febrile illness.

Further studies with a larger sample size and with the possibility of obtaining paired blood samples from the participants should be encouraged to corroborate our research, which has its limitations.

## 5. Conclusions

Our findings revealed that 5.79% of patients with suspected dengue and negative serology for the virus had evidence of infection by pathogenic *Leptospira* spp. This fact may imply under-reporting and insufficient to non-specific treatment of leptospirosis in the study areas. Healthcare providers should increase their awareness of leptospirosis and perform syndromic surveillance to identify the etiologic agent causing the acute febrile illness correctly. Leptospirosis should always be considered in the differential diagnosis of acute febrile illnesses, even in an urban setting. Further in-depth investigations are necessary to diagnose leptospirosis in order to prevent this disease from going unreported with insufficient and non-specific treatment. These results demonstrate the importance of including investigations for *Leptospira* infections in all cases of febrile illness. This will support appropriate patient screening and antibiotic treatment, reducing clinical manifestations and mortality.

## Figures and Tables

**Table 1:** The frequency of the sociodemographic characteristics of suspected dengue patients whose samples were reactive in serological tests for the diagnosis of leptospirosis, Triângulo Mineiro, MG, Brazil, 2019.

	DPP (Positive)	MAT (Positive)
**Number of reactive samples/number of samples analyzed**	26/449	09/52
**Sex**		
Female	16	07
Male	10	02
**Age (years)**		
0–20	05	01
21–40	08	03
41–60	07	03
60 or older	06	02
**Median age (years)** **(minimum/maximum)**	41 (12/80)	56 (20/80)
**Median collection period (days)** **(minimum/maximum)**	7 (05/12)	9 (12/06)
Municipality of origin		
Uberlândia	18	07
Araguari	06	02
Monte Carmelo	02	00
**Race/skin color**		
White	09	04
Brown	12	02
Black	03	01
Ignored	02	01
**Area**		
Periurban/rural	02	00
Urban	24	09
**Admissions**		
Yes	20	00
No	06	09
**Education**		
Illiterate	02	01
Elementary school	02	01
High school	07	00
Higher education	01	00
Not informed	14	07

**Table 2:** Profile of different serovars of nine positive MAT samples in patients suspected of dengue in Triângulo Mineiro, MG, Brazil, 2019.

Sample	DPP	MAT	MAT Titer/Serovar
**1**	Reactive	Reactive	1:800 Canicola
			1:200 Tarassovi
**2**	Reactive	Reactive	1:100 Grippotyphosa
**3**	Reactive	Reactive	1:200 Bratislava
			1:800 Canicola
**4**	Reactive	Reactive	1:200 Canicola
			1:200 Djasiman
			1:200 Grippotyphosa
			1:200 Hardjoprajitino
			1:200 Hebdomadis
			1:200 Icterohaemorragiae
			1:200 Pomona
**5**	Reactive	Reactive	1:100 Copenhageni
			1:200 Grippotyphosa
			1:200 Hardjoprajitino
			1:200 Hebdomadis
**6**	Reactive	Reactive	1:400 Canicola
**7**	Non-reactive	Reactive	1:100 Grippotyphosa
			1:200 Hardjoprajitino
**8**	Non-reactive	Reactive	1:200 Grippotyphosa
			1:200 Hardjoprajitino
**9**	Non-reactive	Reactive	1:200 Grippotyphosa

## Data Availability

The raw data supporting the conclusions of this article will be made available by the authors on request.
